# Large language model-based screening of substances and their composition from safety data sheets for high-resolution chemical exposure assessment

**DOI:** 10.1038/s41370-026-00917-z

**Published:** 2026-05-13

**Authors:** Daeyeop Lee, Kiyoung Lee, Sewon Lee

**Affiliations:** 1 School of Public Health, Seoul National University, Seoul, Republic of Korea; 2 Environmental Risk Research Division, National Institute of Environment Research, Incheon, Republic of Korea; 3 UCD Centre for Safety and Health at Work, School of Public Health, Physiotherapy, and Sports Science, University College Dublin, Dublin, Ireland

**Keywords:** Safety data sheets, Large language models, Performance, Validation

## Abstract

**Background:**

SDSs provide information on chemical substances in professional and consumer products. Large language models (LLMs) offer a rapid approach to screening chemical information from SDSs.

**Objective:**

This study aimed to validate the performance of LLMs in accurately extracting substance information from SDSs of products.

**Methods:**

Chemical information was extracted from the SDSs of cleaning products using the LLMs ChatGPT-4o and Gemini 2.5 Pro. The performance of the LLMs was evaluated against manually extracted data using precision, sensitivity, and F1 score.

**Results:**

A total of 301 substance–composition combinations across 59 products were included in the validation. The Gemini 2.5 Pro model showed a higher F1 score (1.00) than ChatGPT-4o (0.94).

**Significance:**

LLMs enable high-throughput chemical information extraction from SDSs, reducing the burden of manual screening and supporting large-scale combined-exposure assessments.

**Impact Statement:**

The accurate identification of chemical substances in professional and consumer products is a major challenge in assessing complex chemical exposures in exposure science and epidemiology. This study validated the use of Large Language Models (LLMs) as a novel, rapid, and highly accurate method of extracting high-throughput chemical data from multilingual Safety Data Sheets. Our findings illustrated that LLMs could be effectively used to overcome the labor-intensive and time-consuming limitations of manual data screening. This approach allows high-throughput analysis, enabling comprehensive combined-exposure assessments in large-scale epidemiological and exposure assessment studies. Additionally, as LLMs can extract data in different languages, they can facilitate international research collaboration.

## Introduction

Professional and consumer products are a major source of daily chemical exposure. According to the United Nations Environment Program, global chemical sales are projected to increase from €3.47 trillion in 2017 to €6.6 trillion by 2030 [1]. These products contain numerous chemicals, resulting in repeated exposure among users. Chemical exposure is associated with various health hazards, including respiratory, reproductive, and cardiovascular disorders, and cancers [2]. In 2019, the World Health Organization estimated that there were 269,500 deaths due to exposure to several chemicals, including lead, pesticides, occupational carcinogens, and occupational particles, across European regions [2].

Cleaning products are frequently used indoors, leading to repeated exposure of users to various chemicals [3–5]. Previous studies have reported the use of cleaning products in most households [6–8]. Beyond domestic use, people are exposed to complex mixtures of cleaning agents in various occupational settings, extending well-documented risks beyond healthcare environments [5, 9, 10]. Such products contain numerous chemicals, some of which are toxic [11]. Consequently, professional cleaners [12], non-professional cleaners [13], and healthcare workers [10] are at a high risk of exposure to respiratory diseases such as asthma. Recently, a meta-analysis showed a 50% higher risk for asthma among occupational cleaners than among non-cleaners [14].

Despite this established association, one of the main limitations was the identification of individual substances in the products, and of specific causal agents [15]. Epidemiological studies have often assessed exposure to cleaning products based on the self-reported use of products [16] or by using job-related exposure matrices as a proxy for cleaning chemical exposure [17]. However, these methods make it difficult to determine the actual level of exposure and the specific substances involved.

Safety data sheets (SDSs) provide comprehensive information on chemical substances in products and have been used in epidemiological studies to investigate exposure to cleaning agents [18]. SDSs are standardized documents produced by manufacturers and suppliers to communicate hazard and composition information [11, 19].

Within the European Union, the Registration, Evaluation, Authorisation and Restriction of Chemicals (REACH) Regulation (Article 31) mandates the provision of SDSs primarily for professional and industrial users rather than the general public [19]. Therefore, SDS-based extraction is particularly relevant to occupational exposure assessment. Nevertheless, SDSs may also serve as a useful proxy for exposure characterization in the case of dual-use products marketed to both professional and consumer users.

Manual screening of substances across large numbers of SDSs remains time-consuming and labor-intensive, limiting its feasibility for large-scale occupational exposure assessment. To address this limitation, automated data-mining approaches have been developed [20–23]. Although these methods can reduce manual effort, they often require substantial programming expertise and technical infrastructure [24], which may limit their accessibility to users without advanced programming expertise.

Large language models (LLMs), trained on extensive textual data, have demonstrated strong performance in natural language processing tasks such as question answering and structured data extraction [22]. However, the accuracy of LLMs in extracting structured substance and composition information from SDSs has not yet been systematically validated.

This study aimed to validate the performance of LLMs in accurately extracting information on substances and their compositions from SDSs for potential use in chemical exposure assessments for epidemiological or exposome studies in large populations.

## Materials and methods

### Database of substances and their compositions

Air fresheners and cleaning products (e.g., cleaning solvents and laundry detergents) sold in the Irish and South Korean markets were selected for this study because of their wide range of use in both industrial and non-industrial settings. The SDS for each product was obtained from the official manufacturer’s or online supplier’s website between March and June 2025.

Information on individual substances, including chemical names, Chemical Abstracts Service Registry numbers (CAS RN), and composition (% w/w), was extracted from the SDSs using two LLMs: ChatGPT-4o (OpenAI) and Gemini 2.5 Pro (Google). The extraction procedure followed four standardized steps: (1) obtaining SDSs from manufacturer or supplier websites; (2) accessing the selected LLM platform; (3) entering a predefined standardized prompt and uploading the SDS file; and (4) exporting or transferring the structured outputs into a spreadsheet or other database format. When required, the prompt was modified to request output in a machine-readable format (e.g., Excel-compatible table).

An identical standardized prompt was used for both LLMs to ensure methodological consistency. The prompt requested: (1) chemical name, (2) CAS RN, and (3) composition (% w/w) for each substance listed in the SDS. The complete prompts are provided in the Supplementary Information (S1).

### Data processing and analysis

To ensure the accuracy and reliability of the manually extracted data, two researchers independently extracted the chemical names, CAS registry numbers (CAS RN), and composition information from each SDS. The substance information was manually collected, cross-checked, and reviewed by two researchers, who then compiled the entire list of substances and their composition information from SDSs. This information was considered the gold standard for this analysis.

The data extracted by two models were recorded and merged with the gold standard. To generate the combination of CAS RN, minimum and maximum values of composition of each substance, we processed and harmonized the data considering various formats of composition (e.g., “-”, “~”, “≥, ≤” “>,<”).

Finally, the LLM-extracted information was compared with the gold standard based on the CAS RN and the minimum and maximum composition values listed in the SDSs for each substance. Data processing and analysis were conducted using R version 4.4.3 (R Foundation for Statistical Computing, Vienna, Austria).

### Performance assessment of LLMs

The performance of LLMs or machine-learning models(ML) has often been tested by assessment of precision and sensitivity in different areas of research. In this study, the performance of the LLMs was validated against the gold standard, which was manually extracted data. Each single data point comprises a combination of the substances (CAS RNs) and their minimum and maximum compositions. The validation metrics were defined as follows:

True positive (TP): An output where the LLMs correctly extracted all three components, the CAS RN, and its corresponding minimum and maximum composition values.

False positive (FP): An output where the LLMs extracted a CAS RN or minimum and maximum composition values that were incorrect or not present in the gold standard (Type I error).

False negative (FN): An output where the LLMs failed to extract a CAS RN or its corresponding minimum and maximum composition values that were present in the gold standard (Type II error).

Based on these matrices, precision, sensitivity (recall), and F1 scores were calculated as follows:

- Precision (positive predictive value) measures the accuracy of the positive predictions made by the model.
  $${{{\rm{Precision}}}}={{{\rm{TP}}}}/({{{\rm{TP}}}}+{{{\rm{FP}}}})$$
- Sensitivity (recall) measures the model’s ability to identify all the actual positives. It was calculated as:
  $${{{\rm{Sensitivity}}}}={{{\rm{TP}}}}/({{{\rm{FN}}}}+{{{\rm{TP}}}})$$
- The F1 score is the harmonic mean of the precision and sensitivity and provides a single metric to evaluate the overall performance. It was calculated as:

$${{{\rm{F}}}}1{{{\rm{score}}}}=2\times ({{{\rm{precision}}}}\times {{{\rm{sensitivity}}}})/({{{\rm{precision}}}}+{{{\rm{sensitivity}}}})$$

In addition to the predefined validation metrics (precision, sensitivity, and F1 score), hallucination was defined as any LLM output that did not accurately reflect the content of the original SDS and was further classified into three types: (1) incorrect CAS RN assignment, (2) omission of a CAS RN despite correct extraction of the corresponding composition range, and (3) misinterpretation of composition ranges (e.g., incorrect minimum or maximum values).

## Results

### Descriptive characteristics of the selected products

The SDSs of 23 air fresheners (21 and two from Ireland and South Korea, respectively) and 38 cleaning products (27 and 11 from Ireland and South Korea, respectively) were obtained. Air fresheners contained a higher maximum number of substances per product (median: 3; range: 1–43) than cleaning products (median: 3; range: 1–9). A total of 120 and 68 unique substances were identified in the air fresheners and cleaning products, respectively. A total of 173 unique substances were identified across the 23 air fresheners and 38 cleaning products, after removing 15 substances that appeared in both product categories. The most frequently identified substances across all products were isopropanol (CAS RN: 67-63-0) and water (CAS RN: 7732-18-5) in 11 products, and ethanol (CAS RN: 64-17-5) in 10 products. A complete list of substances identified is provided in the Supplementary Information (Table S2).

### Accuracy of LLM-based data extraction

Of the 61 products obtained, two products (Nos. 42 and 45) did not contain any composition information and were excluded, resulting in 59 products for analysis. Of the remaining 321 entries on substances for the 59 products, 20 entries without composition information were further excluded, resulting in a final dataset of 301 substances included in the validation analysis. The F1 score of Gemini 2.5 Pro was 1.00, which means Gemini extracted the information accurately. ChatGPT-4o’s F1 score (0.94) was slightly lower than Gemini’s (Table 1). The ChatGPT-4o and Gemini 2.5 Pro models both exhibited high precision. All substance–composition combinations, including inaccurately extracted combinations by Gemini 2.5 Pro and ChatGPT-4o, are described in Supplementary Table S3.

**Table 1:** Performance metrics of large language model-extracted information on the combination of Chemical Abstracts Service Registry number and composition.

Mode.	Precision	Recall	F1 Score
ChatGPT-4o	0.97	0.91	0.94
Gemini 2.5 Pro	1.00	1.00	1.00

## Discussion

In the present study, the performance of two different LLMs was tested in extracting data on substances and their compositions from SDSs in English and Korean. The Gemini 2.5 Pro model showed slightly better performance than ChatGPT-4o, but the F1 scores of both models were very high (over 0.9). Our findings suggested that these models could help save costs and time when identifying substances for further exposure assessments or epidemiological studies. This study demonstrates the feasibility of applying LLMs to extract data from SDSs and highlights the potential for multilingual extraction, which has not been systematically evaluated.

Our results suggest that LLMs can be used to extract chemicals and their compositions from SDSs rapidly and efficiently in various environmental health studies, particularly in the field of exposure science, because of their scalability and low cost. The basis of chemical information for databases of consumer product chemicals in the US was extracted from SDSs [20]. Such databases can support regulatory agencies by providing structured information on chemical use and exposure potential in consumer products, thereby aiding in the management of chemicals of concern.

Environmental and human biomonitoring provide quantitative measures of exposure and are considered the gold standard for determining chemical exposure. However, these methods are often limited to chemicals with validated analytical methods; specifically, due to technical or analytical constraints, only a small number of substances can be directly monitored, while fieldwork and laboratory analyses require considerable time and cost. Consequently, many chemicals remain unquantified in environmental or biological samples. In such cases, the extraction of chemical information from SDSs could serve as a complementary approach, helping to identify potential exposure sources for under-studied chemicals and to advance exposure assessment by considering co-exposures to chemical mixtures. However, manual extraction or specific coding to extract information when screening large volumes of SDSs often requires greater time costs. For example, the U.S. Environmental Protection Agency (EPA) has developed consumer product ingredient databases based on SDS data [24]. The EPA has since expanded these efforts through the ChemExpo/CPDat platform and provides publicly accessible documentation and data management scripts for automated extraction [25, 26]. Although these approaches are powerful, they may still require substantial computational expertise. LLMs offer a more accessible and user-friendly alternative for researchers and non-experts in exposure science to extract data from SDSs without complex coding. Currently, a company in the USA is using machine-learning models for automated information extraction from SDSs, including products, manufacturers, and supplier names [22]. This company extracted substances and their compositions using machine-learning models with similar precision values (0.98–0.99) but lower recall values (0.70–0.73) compared to manually extracted information [22]. In addition, the extraction process requires complicated procedures, including page filtering, SDS format validation, and specialized architectures with machine-learning models.

The commercial LLMs used in this study (ChatGPT-4o and Gemini 2.5 Pro) have several advantages over bespoke models, including better performance and simpler procedures. First, both ChatGPT-4o (F1 score, 0.94) and Gemini 2.5 Pro models (F1 score, 1.00) showed high precision at extracting the combination of chemicals and their compositions. Second, they require only a simple prompt for intended purposes and not expertise in complex computer languages. Third, the LLMs recognized chemical names in English and Korean. Therefore, they present an application of the SDSs written in various languages. Furthermore, in this study, the LLMs could find the substance information which located in incorrect section, on Section 2 (hazard identification), but not in Section 3 (composition/information on ingredients).

The lower performance of ChatGPT-4o observed in this study may be attributed to challenges in performing optical character recognition (OCR) on image-based SDS documents [25]. In the OCR-processed SDSs (n = 5), 14 composition entries showed complete extraction failure, as neither CAS RN nor composition range information was extracted. In addition, a total of 14 GPT-related hallucination cases were identified, including four instances of incorrect CAS RN generation (e.g., substitution of 64-17-5 with 56539-66-3), seven instances of CAS RN omission despite its presence in the source document, and three instances of composition range misinterpretation (e.g., extraction of “>10–100” instead of “≥1– < 10”). OCR converts text images into machine-readable text, which may cause potential hallucinations in LLM models [22]. We therefore added the text “Inform if the attached SDSs were OCR-processed, and do not show me the results,” to the prompt. The model consequently presented the text “As requested, I will not display the extracted table.” Filtering OCR-processed SDSs may help to improve the accuracy of data extraction.

This study validated the high performance of two different LLMs in extracting data from SDSs, despite errors such as hallucinations and the readability of OCR-processed SDSs. This demonstrates that these tools are feasible for rapidly identifying several substances in a number of consumer/professional products, which is a critical step in toxicological and epidemiological studies. Future studies could apply these LLMs to conduct advanced exposure assessments of individual cleaning agents and to identify complex chemical exposures across diverse populations, including both professional cleaning workers and the general population.

## Supplementary information

Supplementary 1

Supplementary 2 Table S2

Protected view

## Data Availability

The data supporting the findings of this study are provided in the Supplementary Information files accompanying this article.
