# Supplementary material for: Large language model-based screening of substances and their composition from safety data sheets for high-resolution chemical exposure assessment
Source: J Expo Sci Environ Epidemiol. 2026 May 13;36(6):1179–82. doi: 10.1038/s41370-026-00917-z (PMC13727659; doi:10.1038/s41370-026-00917-z)
Supplement: Supplementary file 1 [file 41370_2026_917_MOESM1_ESM.docx]

**Supplementary 1 Prompt to extract chemical name, CAS Registry number, and composition.**

Please extract the chemical name, CAS registration number, and ingredient (% w/w) for each product from Section 3 (Ingredients/Ingredient Information) of the attached SDS file and display them in this table under the columns "Product Name" / "Substance Name" / "Chemical_CasRN" / "Composition (% w/w)". If the SDS is OCR-protected or image-based, do not display the data in a structured table. Instead, please provide an exact citation from Section 3 where the data is mentioned. This is crucial for assessing chemical identity and potential health hazards.
