# Supplementary material for: Large language model-based screening of substances and their composition from safety data sheets for high-resolution chemical exposure assessment
Source: J Expo Sci Environ Epidemiol. 2026 May 13;36(6):1179–82. doi: 10.1038/s41370-026-00917-z (PMC13727659; doi:10.1038/s41370-026-00917-z)
Supplement: Supplementary file 2 [file 41370_2026_917_MOESM2_ESM.docx]

**Supplementary 2 Table S2. The list of substances in cleaning products**

| **No** | **CAS RN** | **Chemical Name** |
| --- | --- | --- |
| 1 | 101-84-8 | diphenyl ether |
| 2 | 101-86-0 | alpha-hexylzimtaldehyd |
| 3 | 102-71-6 | Triethanolamine |
| 4 | 103-95-7 | cyclamen aldehyde |
| 5 | 104-21-2 | p-anisyl acetate |
| 6 | 104-54-1 | cinnamyl alcohol |
| 7 | 104-55-2 | cinnamal |
| 8 | 104-67-6 | undecan-4-olid |
| 9 | 105-87-3 | geranyl acetate |
| 10 | 106-22-9 | citronellol |
| 11 | 106-23-0 | citronellal |
| 12 | 106-24-1 | geraniol |
| 13 | 106-72-9 | dimethyl heptenal |
| 14 | 106-97-8 | butane |
| 15 | 107-98-2 | 1-methoxypropan-2-ol |
| 16 | 1093653-57-6 | 4-(heptyloxy)-3-methylbutanal |
| 17 | 110-41-8 | methylundecanal |
| 18 | 111-76-2 | 2-butoxyethanol |
| 19 | 112-31-2 | decanal |
| 20 | 112-44-7 | undecanal |
| 21 | 112-53-8 | lauryl alcohol |
| 22 | 112-54-9 | lauraldehyde |
| 23 | 112926-00-8 | Silicon dioxide |
| 24 | 115-95-7 | linalyl acetate |
| 25 | 118-58-1 | benzyl salicylate |
| 26 | 12001-26-2 | mica muscovite |
| 27 | 120-51-4 | benzyl benzoate |
| 28 | 1205-17-0 | methylenedioxyphenyl methylpropanal |
| 29 | 1222-05-5 | hexahydrohexamethyl cyclopentabenzopyran |
| 30 | 122-40-7 | 2-benzylidenheptanal |
| 31 | 123-11-5 | anisaldehyde |
| 32 | 123-68-2 | allyl caproate |
| 33 | 124-13-0 | octanal |
| 34 | 124-19-6 | nonanal |
| 35 | 127-51-5 | 3-methyl-4-(2,6,6 trimethyl-2-cyclohexen 1-yl)-3-buten-2-one |
| 36 | 127-91-3 | beta-pinene |
| 37 | 1300-72-7 | sodium xylenesulfonate |
| 38 | 1305-78-8 | calcium oxide |
| 39 | 1310-58-3 | potassium hydroxide |
| 40 | 1310-73-2 | sodium hydroxide |
| 41 | 1335-46-2 | methyl ionone |
| 42 | 1337-83-3 | undecenal |
| 43 | 13463-67-7 | titanium dioxide |
| 44 | 138-86-3 | Dipenten |
| 45 | 1392325-86-8 | 7,7,8,9,9-pentamethyl-6,6a,7,8,9,9a-hexahydro-5h-cyclopenta[h]quinazoline |
| 46 | 139-89-9 | Trisodium 2-(carboxylatomethyl(2-hydroxyethyl)amino)ethyliminodi(acetate) |
| 47 | 140-11-4 | benzyl acetate |
| 48 | 141-43-5 | 2-aminoethanol |
| 49 | 142-19-8 | allyl heptanoate |
| 50 | 144-55-8 | sodium hydrogencarbonate |
| 51 | 14765-30-1 | 2-sec-butylcyclohexanone |
| 52 | 15763-76-5 | benzenesulfonic acid, (1-methylethyl)-, sodium salt |
| 53 | 160875-66-1 | poly(oxy-1,2-ethanediyl), a-(2-propylheptyl)-w-hydroxy- |
| 54 | 16409-43-1 | isobutenyl methyltetrahydropyran |
| 55 | 1643-20-5 | Dodecyldimethylamine oxide |
| 56 | 18127-01-0 | 3-(4-tert butylphenyl)propionald ehyde |
| 57 | 18479-51-1 | 2,6-dimethyl-7-octen-2-ol |
| 58 | 18479-58-8 | 2,6-dimethyloct-7-en-2-ol |
| 59 | 20018-09-1 | diiodomethyl-ptolylsulfone |
| 60 | 20298-69-5 | 2-t-butylcyclohexyl acetate |
| 61 | 2248118-60-5 | (e)-1-((3,6 and 4,6)-dimethylcyclohex-3-en-1-yl)-2-methylpent-1-en-3-one |
| 62 | 22629-49-8 | tridec-2-enenitrile |
| 63 | 23787-90-8 | isolongifolanone |
| 64 | 24937-78-8 | ethylene-vinyl acetate polymer |
| 65 | 25265-71-8 | oxydipropanol |
| 66 | 26183-52-8 | deceth-n |
| 67 | 2634-33-5 | 1,2-benzisothiazolin-3-one |
| 68 | 2705-87-5 | allyl cyclohexylpropionate |
| 69 | 27176-87-0 | Dodecylbenzenesulphonic acid |
| 70 | 27939-60-2 | dimethyl-3-cyclohexene-1-carbaldehyde |
| 71 | 31906-04-4 | 4-(4-hydroxy-4 methylpentyl)cyclohex 3-enecarbaldehyde |
| 72 | 32210-23-4 | 4-tert-butylcyclohexyl acetate |
| 73 | 3228-02-2 | 4-isopropyl-m-cresol |
| 74 | 32388-55-9 | acetylcedrene |
| 75 | 32669-00-4 | ethanone, 1-(3-cycloocten-1-yl) |
| 76 | 33704-61-9 | dihydro pentamethylindanone |
| 77 | 33885-52-8 | tetramethylbicyclo- 2-heptene-2-propion aldehyde |
| 78 | 34398-05-5 | c15 pareth-n |
| 79 | 34590-94-8 | (2-methoxymethylethoxy)propanol |
| 80 | 38888-98-1 | diphenylethane |
| 81 | 4180-23-8 | anethole |
| 82 | 470-82-6 | eucalyptol |
| 83 | 471-34-1 | calcium carbonate |
| 84 | 491-07-6 | isomenthone |
| 85 | 50-00-0 | Formaldehyde |
| 86 | 5064-31-3 | Trisodium nitrilotriacetate |
| 87 | 5131-66-8 | 1-butoxypropan-2-ol |
| 88 | 52474-60-9 | methyl-methylpentenylcyclohexene-1-carbaldehyde |
| 89 | 5392-40-5 | citral |
| 90 | 54464-57-2 | tetramethyl acetyloctahydronaphthalenes |
| 91 | 556-67-2 | octamethylcyclotetrasiloxane [d4] |
| 92 | 56539-66-3 | 3-methoxy-3-methylbutan-1-ol |
| 93 | 57378-68-4 | delta-damascone |
| 94 | 57-55-6 | monopropylene glycol |
| 95 | 58430-94-7 | trimethylhexyl acetate |
| 96 | 586-62-9 | terpinolene |
| 97 | 5949-29-1 | citric acid, monohydrate |
| 98 | 5989-27-5 | d-limonene |
| 99 | 60-12-8 | phenethyl alcohol |
| 100 | 61788-85-0 | peg-40 hydrogenated castor oil |
| 101 | 61792-11-8 | 3,7-dimethylnona-2, 6-dienenitrile |
| 102 | 62099-15-4 | disodium (hydroxyethyl)ethyl enediamine diacetate |
| 103 | 6259-76-3 | hexyl salicylate |
| 104 | 63500-71-0 | isobutyl methyl tetrahydropyranol |
| 105 | 64-02-8 | tetrasodium ethylene diamine tetraacetate |
| 106 | 64-17-5 | ethyl alcohol |
| 107 | 64-17-5/84-66-2 | sd alcohol 39-c (alcohol denat.) |
| 108 | 64-18-6 | formic acid |
| 109 | 64742-47-8 | hydrocarbons, c13-c15, nalkanes, isoalkanes, cyclics, < 2% aroma |
| 110 | 66-25-1 | hexanal |
| 111 | 6728-26-3 | trans-2-hexanal |
| 112 | 67-56-1 | methyl alcohol |
| 113 | 67-63-0 | isopropyl alcohol |
| 114 | 67634-00-8 | isoamyl allylglycolate |
| 115 | 67634-15-5 | ethyl 2,2-dimethylhydrocinnamal |
| 116 | 676532-44-8 | cyclopropanecarboxylic acid,2-methyl-2-[(1,2,4-trimethyl-2-penten-1-yl)oxy]propyl ester |
| 117 | 67801-20-1 | 3-methyl-5-(2,2,3-trimethyl-3-cyclopente n-1-yl)pent-4-en-2-ol |
| 118 | 68002-97-1 | c10-16 pareth |
| 119 | 68039-49-6 | 2,4-dimethyl-3-cyclohexene carboxaldehyde |
| 120 | 68184-04-3 | mea-laureth sulfate |
| 121 | 68213-23-0 | fattyalcohol ethoxylates =/< c15 and =/< 5eo |
| 122 | 68391-01-5 | quaternary ammonium compounds, benzyl-c12-18-alkyldimethyl, chlorides |
| 123 | 68411-30-3 | sodium c10-13 alkyl benzenesulfonate |
| 124 | 68424-85-1 | quaternary ammonium compounds, benzyl-c12-16-alkyldimethyl, chlorides |
| 125 | 68424-95-3 | quaternary ammonium compounds, di-c8-10-alkyldimethyl, chlorides |
| 126 | 68439-45-2 | alcohols, c6-12, ethoxylated |
| 127 | 68439-50-9 | c10-16 pareth |
| 128 | 68439-57-6 | Sulfonic acids, C14-16-alkane hydroxy and C14-16-alkene, sodium salts |
| 129 | 68551-12-2 | alcohols, c12-16, ethoxylated |
| 130 | 68585-34-2 | sodium laureth sulfate |
| 131 | 68608-82-2 | benzene, ethylenated, byproducts from |
| 132 | 68891-38-3 | sodium laureth sulfate |
| 133 | 68912-13-0 | tricyclodecenyl propionate |
| 134 | 68987-42-8 | benzene, ethylenated, residues |
| 135 | 68989-01-5 | quaternary ammonium compounds, benzyl-c12-18-alkyldimethyl, salts with 1,2-benzisothiazol-3(2h)-one 1,1-dioxide (1:1) |
| 136 | 69011-36-5 | alcohols, c13, branched, ethoxylated |
| 137 | 71077-31-1 | 4,8-dimethyl-4,9-decadienal |
| 138 | 74-98-6 | propane |
| 139 | 7681-52-9 | sodium hypochlorite |
| 140 | 7732-18-5 | water |
| 141 | 7757-82-6 | sodium sulphate |
| 142 | 77-83-8 | ethyl methylphenylglycidate |
| 143 | 7786-30-3 | Magnesium chloride |
| 144 | 77-92-9 | citric acid |
| 145 | 78-69-3 | tetrahydrolinalool |
| 146 | 78-70-6 | linalool |
| 147 | 78-70-6/126-91-0 | linalool |
| 148 | 79-77-6 | ionone |
| 149 | 8000-34-8 | eugenia caryophyllus leaf oil |
| 150 | 8000-41-7 | terpineol |
| 151 | 8000-42-8 | carum carvi fruit oil |
| 152 | 8002-74-2 | paraffin waxes and hydrocarbon waxes |
| 153 | 8028-48-6 | Orange, sweet, ext. |
| 154 | 80-54-6 | 2-(4-tert-butylbenzyl)propionaldehyde |
| 155 | 80-56-8 | pinene |
| 156 | 85480-55-3 | mea-c10-13 alkyl benzenesulfonate |
| 157 | 85536-14-7 | dodecylbenzenesulphonic acid |
| 158 | 86115-11-9 | pentamethylheptenone |
| 159 | 87-44-5 | beta-caryophyllene |
| 160 | 88-41-5 | 2-tert-butylcyclohexyl acetat |
| 161 | 89-80-5 | trans-menthone |
| 162 | 9002-92-0 | dodecan-1-ol, ethoxylated |
| 163 | 9004-82-4 | 2-dodecoxyethyl hydrogen sulfate |
| 164 | 91-64-5 | coumarin |
| 165 | 91995-81-2 | fatty acids, (c=10-20) and (c=16-18)-unsated., reactionproducts with triethanol amine, di-me sulfate-quaternized |
| 166 | 97-53-0 | eugenol |
| 167 | 97-54-1 | isoeugenol |
| 168 | 98-55-5 | terpineol |
| 169 | 99-49-0 | carvone |
| 170 | 99-85-4 | gamma-terpinene |
| 171 | 99-86-5 | alpha-terpinene |
| 172 | 99-87-6 | p-cymene |
| 173 | Mixture | fragrance |
